# Galectin-3 as a prognostic biomarker in haemodialysis patients with preserved or mildly reduced ejection fraction

**DOI:** 10.1093/ckj/sfaf306

**Published:** 2025-10-07

**Authors:** Sei Hong Min, Insoo Kim, Jung Nam An, Hyung-Seok Lee, Sung Gyun Kim, Jwa-Kyung Kim

**Affiliations:** Department of Internal Medicine and Kidney Research Institute, Hallym University Sacred Heart Hospital, Anyang, Korea; Department of Internal Medicine and Kidney Research Institute, Hallym University Sacred Heart Hospital, Anyang, Korea; Department of Internal Medicine and Kidney Research Institute, Hallym University Sacred Heart Hospital, Anyang, Korea; Department of Internal Medicine and Kidney Research Institute, Hallym University Sacred Heart Hospital, Anyang, Korea; Department of Internal Medicine and Kidney Research Institute, Hallym University Sacred Heart Hospital, Anyang, Korea; Department of Internal Medicine and Kidney Research Institute, Hallym University Sacred Heart Hospital, Anyang, Korea

**Keywords:** biomarker, galectin-3, heart failure, haemodialysis, preserved ejection fraction

## Abstract

**Background:**

Risk prediction for heart failure (HF) and mortality in haemodialysis (HD) patients with preserved or mildly reduced left ventricular ejection fraction (LVEF) is challenging, as conventional markers often underperform. Galectin-3, a biomarker of fibrosis and inflammation, may offer prognostic value, but its utility in this population remains unclear.

**Methods:**

We prospectively enrolled 244 incident HD patients with an LVEF ≥40%. The primary outcome was a composite of cardiovascular (CV) death or acute HF hospitalization. Secondary outcomes included all-cause mortality and individual primary outcome components. Predictive performance was assessed by comparing a baseline clinical model with models incorporating brain natriuretic peptide (BNP) and/or galectin-3, using the area under the curve (AUC), net reclassification improvement (NRI) and integrated discrimination improvement (IDI).

**Results:**

The mean baseline LVEF was 59.5% and the median BNP and galectin-3 levels were 357.5 pg/ml and 34.3 ng/ml, respectively. Over a median follow-up of 37 months, high galectin-3 levels (≥34.3 ng/ml) were associated with higher incidence rates of the primary outcome [14.09 versus 5.87 per 100 person-years (PY)] and all-cause mortality (14.09 versus 6.33 per 100 PY). In multivariate analysis, elevated galectin-3 independently predicted the composite outcome {hazard ratio [HR] 2.16 [95% confidence interval (CI) 1.17–4.00], *P* = .014], acute HF [HR 2.11 (95% CI 1.07–4.14), *P* = .033] and all-cause mortality [HR 1.90 (95% CI 1.04–3.55), *P* = .043]. Adding galectin-3 to the clinical model significantly improved discrimination (AUC 0.735 versus 0.678; ΔAUC 0.057; *P* = .025) and reclassification metrics (NRI 0.565; IDI 0.058; both *P* < .001). In contrast, BNP addition did not significantly enhance prediction.

**Conclusion:**

In incident HD patients with an LVEF ≥40%, elevated galectin-3 was an independent predictor of CV death or acute HF events and provided incremental prognostic value beyond conventional clinical parameters and BNP. These findings support galectin-3 as a complementary biomarker for enhanced risk stratification in this population.

## GRAPHICAL ABSTRACT

**GRAPHICAL ABSTRACT:**
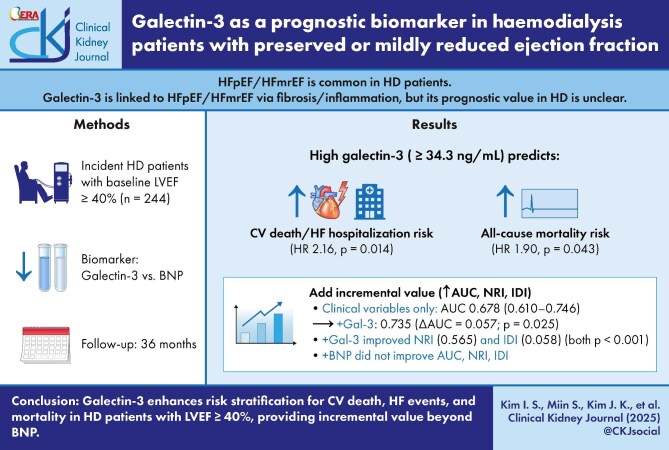

KEY LEARNING POINTS **What was known:**

- Heart failure (HF) with preserved or mildly reduced ejection fraction is common in haemodialysis (HD) patients, but diagnosis and risk stratification are challenging due to overlapping symptoms and the limited specificity of B-type natriuretic peptide (BNP) in end-stage kidney disease.

**This study adds:**

- Elevated galectin-3 was independently associated with higher risks of cardiovascular (CV) death or acute HF hospitalization and all-cause mortality.
- Incorporating galectin-3 into a conventional clinical model improved prediction accuracy and risk reclassification beyond that provided by BNP.

**Potential impact:**

- Galectin-3 may serve as a complementary biomarker to enhance CV risk stratification in HD patients with preserved systolic function, a setting where traditional markers often underperform.

## INTRODUCTION

Heart failure (HF) is highly prevalent in patients with end-stage kidney disease (ESKD) and represents a major contributor to morbidity and mortality. HF is typically classified into three phenotypes based on left ventricular ejection fraction (LVEF): reduced (HFrEF), mildly reduced (HFmrEF) and preserved (HFpEF). Among ESKD patients, HFpEF and HFmrEF are the dominant phenotypes [1, 2]. However, conventional diagnostic and prognostic tools such as LVEF and B-type natriuretic peptide (BNP) often provide limited clinical utility in this population [3–5].

This limitation stems from the unique pathophysiology in HFpEF and HFmrEF, which are characterized by myocardial fibrosis, chronic inflammation and ventricular stiffening and may not be adequately captured by traditional markers [1, 6]. In addition, BNP levels are frequently elevated in ESKD irrespective of cardiac function, due to impaired renal clearance and persistent volume expansion [7–9]. Furthermore, symptoms such as dyspnoea and fluid retention are commonly observed in ESKD patients due to uraemia, making it even more challenging to distinguish HF from volume-related symptoms [10].

Despite the predominance of HFpEF and HFmrEF in haemodialysis (HD) patients, these phenotypes have been underrepresented in prognostic research. Major guidelines such as the Kidney Disease Outcomes Quality Initiative do not specify biomarkers for risk stratification beyond standard echocardiography [11]. This underscores the need for novel biomarkers that more accurately reflect the underlying fibrotic and inflammatory mechanisms of HF in this setting.

Galectin-3, a β-galactoside-binding lectin secreted by activated macrophages, has emerged as a promising biomarker of cardiac fibrosis and inflammation [12, 13]. Unlike natriuretic peptides, which primarily reflect myocardial stretch and haemodynamic stress, galectin-3 may reflect underlying fibrotic and inflammatory processes contributing to cardiac dysfunction [14]. Elevated galectin-3 levels have been associated with adverse cardiovascular (CV) outcomes in the general population as well as HF patients [15–17]. Even in ESKD patients, recent evidence has demonstrated that higher galectin-3 is linked to increased mortality [18]. However, its incremental prognostic value relative to BNP, particularly in HD patients with preserved or mildly reduced LVEF, remains insufficiently explored.

This prospective cohort study investigated the long-term prognostic significance of galectin-3 in incident HD patients with a baseline LVEF ≥40% and compared its predictive performance with a baseline clinical model, both with and without the combination of BNP.

## MATERIALS AND METHODS

### Study population and design

**Figure 1:**
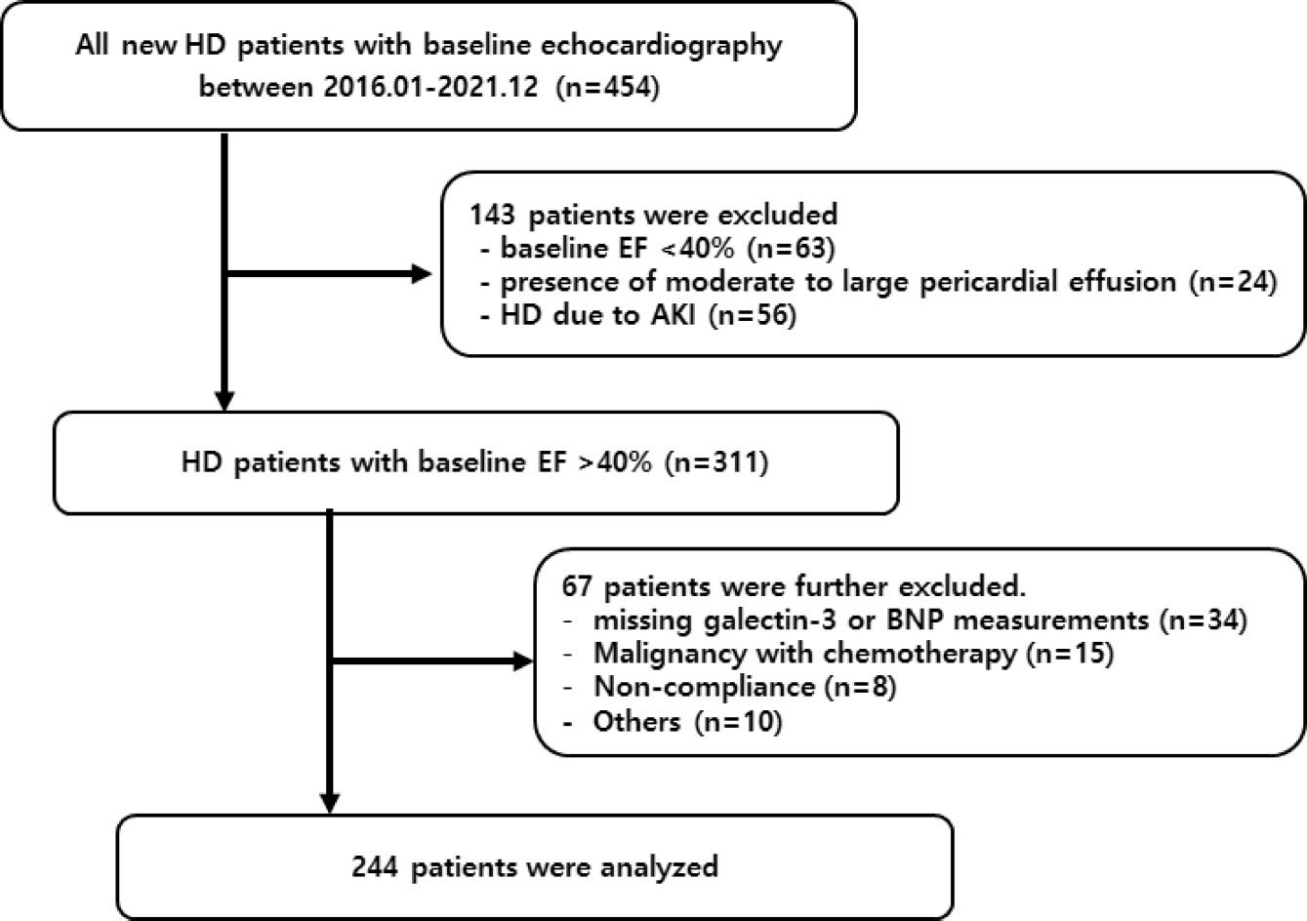
Flow diagram of study population selection.

This prospective, longitudinal cohort study was conducted at Hallym University Sacred Heart Hospital (Anyang, Korea) between January 2016 and December 2021. Among 454 incident HD patients who underwent baseline echocardiography, 143 were excluded: baseline LVEF <40% (*n* = 63), presence of moderate to large pericardial effusion (*n* = 24) or starting HD for acute kidney injury (AKI) rather than ESKD (*n* = 56). Of the remaining 311 patients with an LVEF ≥40%, an additional 67 were excluded due to missing galectin-3 or BNP data, active malignancy under treatment or insufficient follow-up, resulting a final study cohort of 244 patients (Fig. 1).

Patients with HFrEF (LVEF <40%) were excluded, as their pathophysiological mechanisms and biomarker profiles differ substantially from those of HFpEF and HFmrEF. Galectin-3, a marker of myocardial fibrosis and inflammation, is considered more pathophysiologically relevant in HFpEF and HFmrEF. Given the diagnostic challenges associated with HFpEF or HFmrEF in patients with ESKD, enrolment was based solely on preserved or mildly reduced LVEF (≥40%), regardless of clinical HF symptoms. The study protocol was approved by the Institutional Review Board of Hallym University Sacred Heart Hospital (IRB No. 2015-I113) and written informed consent was obtained from all participants. The study was conducted in accordance with the principles of the Declaration of Helsinki.

Baseline demographic and clinical characteristics were recorded at enrolment. All patients underwent thrice-weekly HD (or twice weekly in selected cases), with each session lasting 3.5–4 h.

### Biomarker measurement

Venous blood samples were collected before the first HD session. Laboratory tests included complete blood count, haemoglobin, albumin, calcium, phosphorus, lipids, blood urea nitrogen, creatinine and high-sensitivity C-reactive protein (hsCRP). The calcium–phosphorus product and neutrophil:lymphocyte ratio (NLR) were calculated.

BNP levels were measured at HD initiation using a chemiluminescent microparticle immunoassay on the Alinity i platform (Abbott Laboratories, Abbott Park, IL, USA) as part of routine clinical care. hsCRP levels were determined by a latex-enhanced immunoturbidimetric assay on the COBAS-8000 platform (Roche Diagnostics, Mannheim, Germany).

Serum galectin-3 levels were assessed immediately before the first dialysis session using a commercially available enzyme-linked immunosorbent assay kit (R&D Systems, Minneapolis, MN, USA), following the manufacturer’s instructions. The intra-assay and interassay coefficients of variation were 4.3% and 6.1%, respectively.

### Echocardiographic data

Baseline transthoracic echocardiography was performed after the initiation of maintenance HD once adequate volume control had been achieved and overt oedema had resolved, in order to minimize the influence of acute volume overload on cardiac measurements, using a Vivid 7 ultrasound system (GE Vingmed, Horten, Norway) with a 2.5-MHz probe.

Studies were conducted by cardiologists blinded to clinical data. Standard M-mode, two-dimensional and Doppler measurements were obtained following American Society of Echocardiography guidelines. LVEF was calculated using the modified Simpson’s biplane method. Additional parameters such as the E:e′ ratio and left atrial volume index (LAVI) were recorded when available. Left ventricle geometry was classified as normal, concentric hypertrophy, eccentric hypertrophy or concentric remodelling. The presence of left ventricular hypertrophy (LVH) was also noted.

### Outcomes

The primary outcome was a composite of CV death, defined as death attributable to myocardial infarction (MI), HF or sudden cardiac death (SCD); and acute HF events, defined as emergency room visits or hospitalizations for decompensated HF. Acute HF events required compatible symptoms (e.g. dyspnoea, peripheral oedema), radiographic evidence of pulmonary congestion on chest X-ray or computed tomography and a BNP level ≥200 pg/ml. Patients presenting with dyspnoea but a BNP level <200 pg/ml were not classified as having an HF event [8, 19].

Secondary outcomes included all-cause mortality and the individual components of the primary outcome. Follow-up continued through December 2024. Twenty-four patients (9.8%) were transferred to other hospitals and censored at the last clinical contact; no other losses to follow-up occurred.

### Statistical analysis

All statistical analyses were performed using R version 4.3.1 (R Foundation for Statistical Computing, Vienna, Austria) or SPSS version 24 (IBM, Armonk, NY, USA). Two-sided *P*-values <.05 were considered statistically significant. Continuous variables were expressed as mean ± standard deviation (SD) or median [interquartile range (IQR)] and compared using the Student’s *t*-test or Mann–Whitney U test. Categorical variables were compared using the chi-squared or Fisher’s exact test. Correlations between galectin-3 and clinical parameters were assessed using Pearson’s or Spearman’s correlation coefficients. Patients were stratified into high and low galectin-3 groups using the median value (34.3 ng/ml) and additionally analysed in quartiles to explore dose–response associations. Kaplan–Meier survival curves were used for time-to-event analysis, with comparisons via the logrank test. Cox proportional hazards models estimated hazard ratios (HRs) and 95% confidence intervals (CIs).

To evaluate the incremental prognostic value of galectin-3, we compared a baseline clinical model with models incorporating BNP, galectin-3 or both. Discriminative ability was assessed using receiver operating characteristics (ROC) curves and differences in the area under the curve (AUC) were tested using DeLong’s method. Net reclassification improvement (NRI) was calculated to quantify the added value of galectin-3 to the baseline clinical model.

## RESULTS

### Baseline clinical and echocardiographic characteristics

A total of 244 incident HD patients with a baseline LVEF ≥40% were included (Fig. 1). The median serum galectin-3 level was 34.3 ng/ml (Supplemental Fig. 1) and patients were divided into high and low galectin-3 groups based on this cut-off. As shown in Table 1, patients in the high galectin-3 group were older (70.2 ± 12.7 versus 67.0 ± 12.6 years; *P* = .040) and had a higher prevalence of pre-existing coronary artery disease (CAD; 28.2% versus 11.7%; *P* = .001). Inflammatory markers such as white blood cell count (WBC), neutrophil count, NLR and hsCRP were all significantly elevated in the high galectin-3 group. They also had higher serum phosphorus and BNP levels and lower high-density lipoprotein cholesterol (HDL-C) levels.

**Table 1:** Baseline characteristics of the study population by galectin-3 group.

		Galectin-3		
Variables	Total (*N* = 244)	Low (<34.3 ng/ml)	High (≥34.3 ng/ml)	*P*-value
Age (years)	68.6 ± 12.5	67.0 ± 12.6	70.2 ± 12.7	.040
Male, *n* (%)	143 (58.6)	66 (55.0)	77 (62.1)	.160
Diabetes, *n* (%)	155 (63.5)	73 (60.8)	82 (66.1)	.234
Pre-existing CAD, *n* (%)	49 (20.1)	14 (11.7)	35 (28.2)	.001
SBP (mmHg)	139.1 ± 24.6	139.1 ± 22.6	139.6 ± 26.6	.917
DBP (mmHg)	77.6 ± 10.5	76.8 ± 10.5	78.4 ± 10.5	.359
BMI (kg/m^2^)	24.7 ± 4.6	24.6 ± 4.9	24.8 ± 4.3	.807
WBC (× 10^3^/μl)	6810 ± 1814	6498 ± 1771	7112 ± 1814	.017
Haemoglobin (g/dl)	9.1 ± 1.7	9.1 ± 1.6	9.1 ± 1.7	.886
Neutrophil (× 10^3^/μl)	4745 ± 1683	4400 ± 1432	5080 ± 1842	.004
Neutrophil/lymphocyte	4.6 ± 3.2	4.0 ± 2.4	5.1 ± 3.7	.017
Serum calcium (mg/dl)	8.1 ± 0.8	8.23 ± 0.80	8.13 ± 0.98	.412
Serum phosphate (mg/dl)	4.8 ± 1.5	4.6 ± 1.2	5.1 ± 1.7	.024
Total cholesterol (mg/dl)	153.5 ± 50.5	148.4 ± 45.8	157.1 ± 54.5	.181
Triglyceride (mg/dl)	135.0 ± 84.5	131.8 ± 87.6	137.89 ± 81.2	.620
HDL cholesterol (mg/dl)	42.6 ± 13.7	45.6 ± 15.2	41.6 ± 12.0	.037
LDL cholesterol (mg/dl)	90.4 ± 34.2	89.7 ± 30.6	91.0 ± 37.3	.791
Albumin (g/dl)	3.4 ± 0.5	3.4 ± 0.5	3.3 ± 0.5	.315
BNP (pg/ml), median (IQR)	357.5 (151.5–1011.81)	279.5 (136.2–816.5)	464.2 (187.8–1165.2)	.006
hsCRP (mg/l), median (IQR)	1.13 (0.56–2.93)	0.92 (0.38–2.26)	1.63 (0.86–4.54)	.003
Echocardiographic parameters				
LVEF (%)	59.5 ± 7.4	60.6 ± 6.6	58.4 ± 8.0	.025
LV geometry				.001
Normal	101 (41.3)	66 (55.0)	35 (29.1)	
Concentric hypertrophy	71 (29.0)	24 (20.0)	47 (39.2)	
Eccentric hypertrophy	44 (18.0)	16 (13.3)	28 (23.3)	
Concentric remodelling	128 (11.4)	14 (11.7)	14 (11.6)	
LVH	115 (47.1)	40 (33.3)	75 (62.5)	<.001
E/e′	13.8 ± 5.5	13.5 ± 5.5	14.1 ± 5.0	.382
LAVI	54.0 ± 15.4	55.6 ± 16.6	52.5 ± 14.1	.134

Data are presented as mean ± SD unless stated otherwise.

LDL: low-density lipoprotein.

Of the total cohort, 26 patients (10.7%) had mildly reduced LVEF (41–49%). The high galectin-3 group had a significantly lower mean LVEF (58.4 ± 8.0% versus 60.6 ± 6.6%; *P* = .025) and a greater prevalence of LVH (62.5% versus 33.3%; *P* < .001), with more frequent concentric or eccentric hypertrophy patterns (Supplemental Fig. 1). No significant group differences were observed in the E/e′ ratio or LAVI between groups.

### Correlation of galectin-3 with clinical parameters

Galectin-3 levels were positively correlated with age (*r* = 0.151, *P* = .018), pre-existing CAD (*r* = 0.202, *P* = .002), WBC (*r* = 0.188, *P* = .008), neutrophil count (*r* = 0.239, *P* = .001), NLR (*r* = 0.158, *P* = .026), hsCRP (*r* = 0.254, *P* = .001) and serum phosphorus (*r* = 0.179, *P* = .005) (Table 2). Galectin-3 was also correlated with baseline BNP levels (*r* = 0.229, *P* < .001). Conversely, it was negatively correlated with HDL-C (r = –0.182, *P *= 0.005), but showed no significant association with LVEF.

**Table 2:** Correlations between galectin-3 vascular risk profiles.

Variables	Age	CAD	WBC	Neutrophils	Phosphorus	hsCRP	BNP	HDL	EF	LVH
Galectin-3	0.151*	0.202**	0.188**	0.239**	0.179**	0.254*	0.229**	−0.182*	−0.083	0.139*
Age		0.157*	−0.059	0.007	−0.175**	0.276*	0.117	−0.028	−0.018	−0.011
CAD			0.096	0.074	−0.104	0.174*	0.132*	−0.106	−0.172**	−0.005
WBC count				0.803**	−0.076	0.203*	−0.016	−0.054	0.043	0.042
Neutrophil					−0.073	0.244*	0.026	−0.081	0.038	0.107
Phosphorus						−0.025	0.104	0.015	−0.057	0.102
hsCRP							0.239**	−0.145	−0.122	0.051
BNP								0.054	−0.313**	0.128
HDL									0.057	0.058
LVEF										−0.046

^*^ *P* < .05, ^**^P < .01.

SBP: systolic blood pressure; DBP: diastolic blood pressure; LDL: low-density lipoprotein; BMD: bone mineral density/.

In contrast, serum BNP was negatively correlated with baseline LVEF (*r* = −0.313, *P* < .001) but showed no association with age, neutrophil count or HDL-C. These findings suggest that galectin-3 and BNP reflect distinct pathophysiological characteristics, fibrosis/inflammation versus haemodynamic stress, in the HD population.

### Galectin-3 and the risk of clinical outcomes

During a median follow-up of 37 months (IQR 13.4–60.3), a total of 62 acute HF events and 84 deaths occurred, of which 30 (35.7%) were attributable to CV causes and 54 (64.3%) to non-CV causes (infection related, *n* = 38; malignancy related, *n* = 8; other, *n* = 8). The median BNP level at the time of acute HF was 1843.0 pg/ml (IQR 746.2–2143.9). The total follow-up time was 5302.6 person-years (PY) in the low galectin-3 group and 5086.1 PY in the high group.

The incidence of the primary composite outcome was significantly higher in the high galectin-3 group [event rate: 46.7% versus 20.5%; incidence rate: 14.09 versus 5.87 per 100 PY; adjusted HR 2.16 (95% CI 1.17–4.00), *P* = .014]. The incidence of acute HF events was also higher in the high galectin-3 group [event rate: 33.6% versus 17.2%; incidence rate: 10.14 versus 4.93 per 100 PY; adjusted HR 2.11 (95% CI 1.07–4.14), *P* = .033]. Similarly, all-cause mortality was more common in the high galectin-3 group [event rate: 46.7% versus 22.1%; incidence rate: 14.09 versus 6.33 per 100 PY; adjusted HR 1.73 (95% CI 1.04–3.55), *P* = .043] (Table 3).

**Table 3:** Incidence rates and multivariable hazard ratios for clinical outcomes by galectin-3 group.

		Primary composite outcomes		All-cause mortality		Acute HF hospitalization	
Variable	Adjusted HR^a^ (95% CI)	*P*-value	Adjusted HR (95% CI)	*P*-value	Adjusted HR (95% CI)	*P*-value	
Galectin-3	High versus low	2.16 (1.17–4.00)	.014	1.90 (1.042–3.55)	.040	2.11 (1.07–4.14)	.033
Age	1 year	1.05 (1.02–1.08)	<.001	1.06 (1.03–1.09)	<.001	1.05 (1.02–1.08)	.005
Pre-existing CAD	Yes	1.10 (0.55–2.22)	.779	1.07 (0.58–2.0)	.817	1.07 (0.63–1.83)	.800
Baseline EF (%)	<50% versus ≥50%	0.43 (0.13–1.37)	.155	1.26 (0.59–2.71)	.543	1.10 (0.55–2.16)	.776
Baseline BNP	1 unit increase	1.01 (0.97–1.02)	.351	1.06 (0.78–1.11)	.685	1.02 (0.84–1.25)	.587
Baseline hsCRP	1 unit increase	1.16 (0.87–1.56)	.299	1.08 (1.01–1.17)	.023	1.04 (1.01–1.15)	.045

^a^Adjusted for age, sex, pre-existing CAD, LVEF, BNP, hsCRP level and galectin-3.

**Figure 2:**
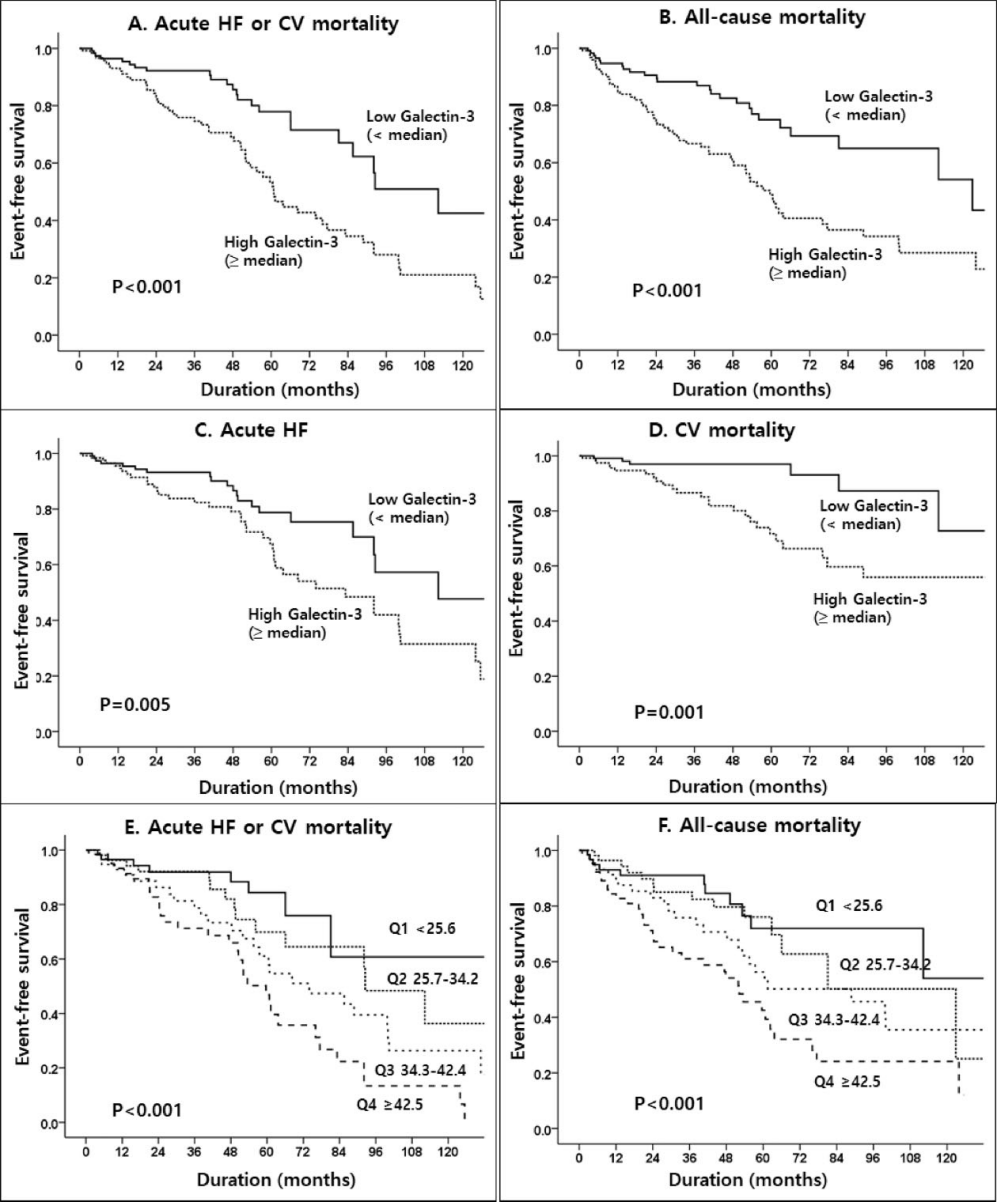
Kaplan–Meier curves for event-free survival by median galectin-3 level. Event-free survival is shown for **(A)** the primary composite outcome (CV death or acute HF hospitalization), **(B)** all-cause mortality and **(C, D)** each component of the primary outcome, stratified by baseline galectin-3 levels (high ≥34.3 ng/ml versus low <34.3 ng/ml). **(E, F)** A dose–response relationship was observed, with progressively higher event rates across galectin-3 quartiles.

Kaplan–Meier curves showed significantly lower event-free survival in the high galectin-3 group for all outcomes (Fig. 2). In multivariable Cox models adjusted for age, pre-existing CAD, LVEF, BNP and hsCRP, both older age and high galectin-3 levels remained independent predictors of the primary composite outcome and all-cause mortality (Table 3).

To further evaluate potential dose–response relationships, galectin-3 levels were categorized into quartiles. Compared with the lowest quartile (Q1), both Q3 [≥34.3 ng/ml; adjusted HR 2.36 (95% CI 1.07–5.23), *P* = .034] and Q4 [≥42.5 ng/ml, adjusted HR 3.20 (95% CI 1.46–7.04), *P* = .004] were significantly associated with an increased risk of the primary outcome. A similar pattern was observed for all-cause mortality, supporting a graded association between galectin-3 levels and adverse outcomes (Fig. 2E and F).

In an exploratory analysis of cause-specific CV mortality (*n* = 30), deaths were evenly distributed across MI/SCD (*n* = 10), HF-related death (*n* = 10) and cerebrovascular accident (CVA)-related death, including both ischaemic and haemorrhagic strokes (*n* = 10). Median galectin-3 levels were highest in the MI/SCD group [49.7 ng/ml (IQR 38.4–63.2)], followed by HF-related [median 47.1 ng/ml (IQR 34.3–53.5)] and CVA-related deaths [median 37.3 ng/ml (IQR 26.5–39.4)]. This pattern suggests a potential association between galectin-3 and ischaemic or vascular inflammatory processes, in addition to myocardial fibrosis (Supplemental Table 1).

### Discriminative performance of galectin-3

To evaluate the prognostic performance of galectin-3, we compared a baseline clinical model [model 1: age, sex, LVEF, SBP, body mass index (BMI), diabetes mellitus (DM) and hsCRP] with models incorporating BNP, galectin-3 or both. The baseline model yielded an AUC of 0.678 (95% CI 0.610–0.746). Adding BNP did not significantly improve discrimination (AUC 0.692; ΔAUC 0.014; *P* = .362), nor did it significantly improve reclassification (NRI 0.148, *P* = .175; IDI 0.009, *P* = .133).

**Figure 3:**
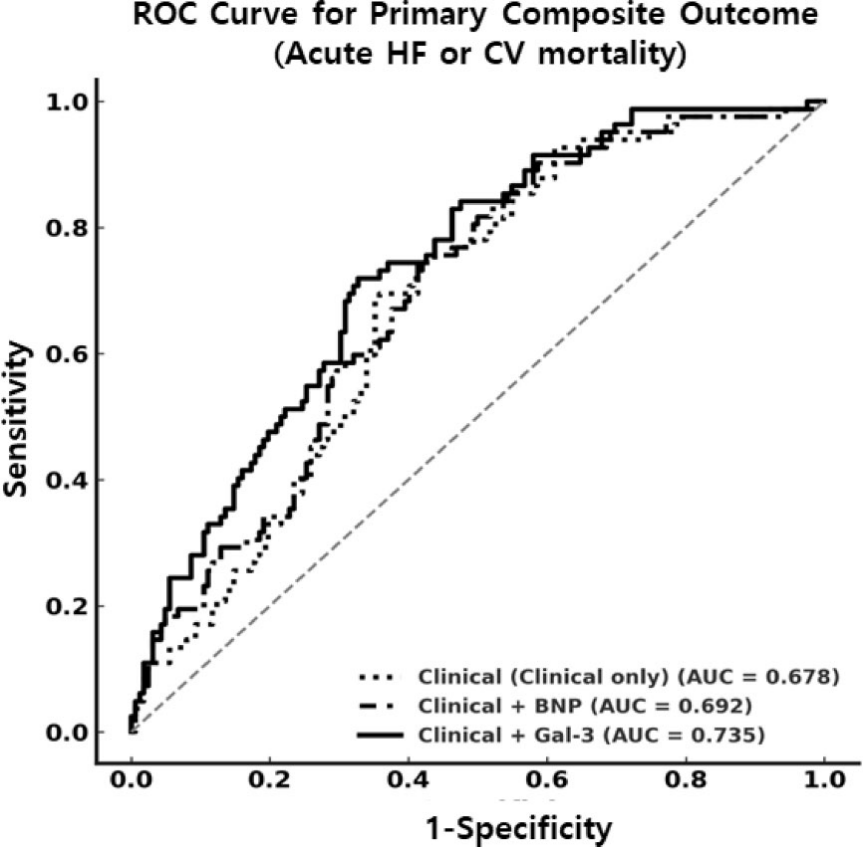
Comparison of predictive performance for the primary composite outcome. ROC curves comparing the discriminative performance of three models for predicting CV death or acute HF hospitalization: a clinical model (age, sex, LVEF, SBP, BMI, DM and hsCRP), the clinical model plus BNP and the clinical model plus galectin-3. The model incorporating galectin-3 yielded the highest AUC, suggesting improved predictive accuracy compared with the others.

In contrast, the addition of galectin-3 significantly improved the AUC (0.735; ΔAUC 0.057; *P* = .025) and yielded significant improvements in reclassification metrics (NRI 0.565, *P* < .001; IDI 0.058, *P* < .001) (Fig. 3). Compared with the clinical + BNP model, the clinical + galectin-3 model demonstrated superior reclassification (NRI 0.430, *P* = .001; IDI 0.042, *P* = .009). Furthermore, the addition of galectin-3 to the clinical + BNP model further improved prognostic performance (NRI 0.577, *P* < .001; IDI 0.048, *P* = .002) (Table 4). These findings support the incremental and complementary prognostic value of galectin-3 beyond conventional clinical variables and BNP in HD patients with preserved or mildly reduced LVEF.

**Table 4:** Comparison of predictive models incorporating BNP and galectin-3 for the primary outcome.

Model	AUC (95% CI)	∆AUC (*P*-value)	NRI (95% CI)	*P*-value (NRI)	IDI (95% CI)	*P*-value (IDI)
Clinical model only (model 1)^a^	0.678 (0.610–0.746)	Reference	Reference	–	Reference	–
Model 1 + BNP	0.692 (0.625–0.760)	0.014 (0.362)	0.148 (−0.062– 0.356)	.175	0.009 (−0.003–0.021)	.133
Model 1 + galectin-3	0.735 (0.672–0.798)	0.057 (0.025)	0.565 (0.328–0.772)	<.001	0.058 (0.028–0.089)	<.001
Model 1 + BNP + galectin-3	0.739 (0.677–0.801)	0.061 (0.021)	0.572 (0.332–0.780)	<.001	0.061 (0.030–0.092)	<.001
Model 1 + BNP versus model 1 + galectin-3		0.043 (0.044)	0.430 (0.184–0.656)	.001	0.042 (0.011–0.072)	.009

^a^Model 1: age, sex, LVEF, SBP, BMI, DM and hsCRP.

## DISCUSSION

In this prospective cohort of incident HD patients with preserved or mildly reduced LVEF (≥40%), elevated serum galectin-3 levels were independently associated with an increased risk of the primary composite outcome, CV death or acute HF hospitalization, as well as all-cause mortality. Galectin-3 showed superior prognostic performance compared with BNP, significantly improving risk discrimination and reclassification when added to a baseline clinical model. In contrast, BNP did not meaningfully enhance model performance. These findings suggest that galectin-3 provides incremental and complementary prognostic value beyond traditional risk factors and BNP in HD patients with non-reduced systolic function.

HF is a common complication in HD patients, with HFpEF or HFmrEF increasingly recognized as the predominant phenotypes [1, 2, 20]. However, diagnosis remains difficult due to overlapping symptoms with volume overload and uraemia and the limited specificity of natriuretic peptides such as BNP in the context of impaired renal clearance [9, 21, 22]. These challenges highlight the need for alternative biomarkers that better reflect the underlying myocardial pathophysiology [6].

Galectin-3, a macrophage-derived lectin involved in myocardial fibrosis and inflammation, contributes to LV remodelling and progressive cardiac dysfunction [14, 23]. It has been associated with adverse outcomes in both HF patients and the general population [18, 24]. A recent meta-analysis reinforced its role in predicting incident HF and all-cause mortality in ESKD patients [18]. By promoting type I collagen synthesis and extracellular matrix accumulation, galectin-3 serves as a biologically relevant biomarker for risk stratification in HD patients with preserved or mildly reduced LVEF [24–26].

In this prospective study, we found that elevated serum galectin-3 levels were significantly associated with an increased risk of the primary composite outcome, CV death or acute HF hospitalization. This association remained robust after adjustment for age, pre-existing CAD, LVEF, baseline BNP and hsCRP. And a clear dose–response relationship was observed, with progressively higher risk across galectin-3 quartiles and the greatest risk in the top quartile (Q4 ≥42.5 ng/ml). Higher galectin-3 levels, whether categorized by quartiles or the median (≥34.3 ng/ml), were consistently associated with worse clinical outcomes. These findings highlight galectin-3 as a clinically meaningful biomarker for risk stratification in HD patients with non-reduced systolic function, a population in which conventional HF assessment tools are often limited. Further large-scale, multicentre studies are warranted to define clinically applicable thresholds.

Galectin-3 also predicted all-cause mortality, consistent with prior meta-analyses. This may reflect the pleiotropic role of galectin-3, which is upregulated not only in cardiac fibrosis but also in systemic processes such as aging, malignancy and neurodegenerative disease [27–29]. In our cohort, infection- and malignancy-related deaths accounted for the majority of non-CV deaths, and median galectin-3 levels in these patients were comparable to those who died from CV causes. These findings align with previous studies by Obokata *et al*. [30] and Hogas *et al*. [31], which demonstrated galectin-3 as an independent predictor of all-cause mortality in HD populations. In contrast, a prior study in a prevalent HD cohort reported no association between galectin-3 and adverse outcomes [32]. That study included patients with a longer dialysis vintage of 4 years and did not exclude those with HFrEF, which may have attenuated the biomarker’s prognostic relevance. In our incident HD cohort, more dynamic changes in cardiac structure, function and fluid status at dialysis initiation may have heightened the prognostic relevance of galectin-3, potentially explaining the discrepant findings.

Another key finding was that galectin-3 outperformed BNP in predicting the composite outcome when added to a baseline clinical model. While BNP provided limited incremental value, galectin-3 significantly improved overall risk discrimination and reclassification. This suggests that galectin-3 offers complementary prognostic information beyond BNP, likely reflecting fibrotic and inflammatory processes not captured by volume-based markers in HD patients. From a clinical perspective, elevated galectin-3 levels may help identify high-risk patients who could benefit from closer follow-up, cardiology referral or targeted cardiac imaging. It may also prompt more careful volume management to prevent HF decompensation. Although not routinely used in dialysis care, galectin-3 could be integrated into multimarker strategies to improve individualized risk stratification.

This study has several limitations. First, acute HF events were adjudicated retrospectively based on clinical symptoms and BNP levels, which may have introduced misclassification. Second, echocardiographic assessment of diastolic function was incomplete in some patients, limiting detailed HFpEF phenotyping. Third, the optimal galectin-3 cut-off remains uncertain and may vary by population. Fourth, objective markers of volume status, such as interdialytic weight gain, ultrafiltration volume or bioimpedance, were not systematically collected, limiting our ability to assess the effect of congestion on biomarker performance. Fifth, only baseline measurements of galectin-3 and BNP were available; serial measurements may provide additional prognostic value, as temporal changes in galectin-3 have been linked to outcomes in HF populations [33, 34]. Lastly, as this was a single-centre study in Korea, the findings may not be generalizable to other HD populations, particularly those in different countries or dialysis settings. External validation in diverse cohorts is needed.

## CONCLUSION

Despite these limitations, our study highlights galectin-3 as a valuable prognostic biomarker in HD patients with preserved or mildly reduced LVEF. It demonstrated superior risk stratification compared with BNP and was independently associated with adverse outcomes, including CV death, acute HF events and all-cause mortality. Galectin-3 may help identify high-risk patients who are not adequately captured by conventional markers, supporting its potential role in personalized risk assessment. Future studies should validate these findings and explore galectin-3-guided strategies in broader HD populations.

## Supplementary Material

sfaf306_Supplemental_Files

## Data Availability

The data underlying this article will be shared upon reasonable request to the corresponding author.
