# Supplementary material for: Galectin-3 as a prognostic biomarker in haemodialysis patients with preserved or mildly reduced ejection fraction
Source: Clin Kidney J. 2025 Oct 7;18(10):sfaf306. doi: 10.1093/ckj/sfaf306 (PMC12556406; doi:10.1093/ckj/sfaf306)
Supplement: sfaf306_Supplemental_Files [file sfaf306_supplemental_files.zip › Revised Supplem Figure 1.pdf]

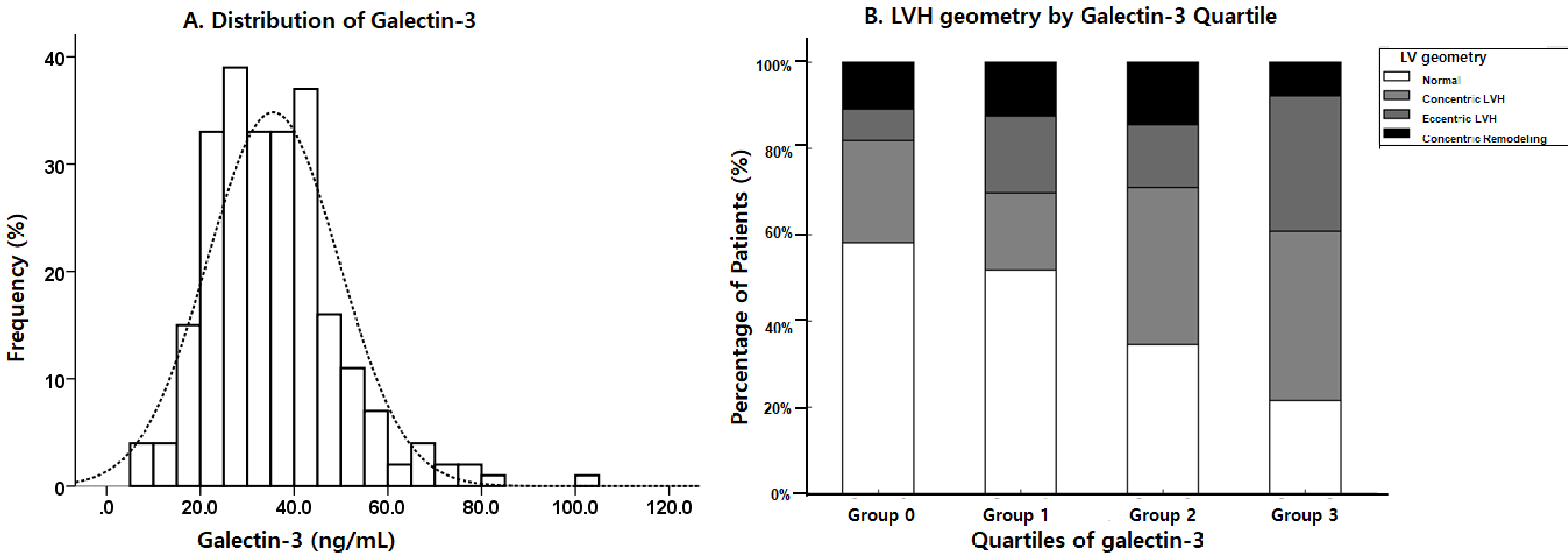

Supplemental Figure 1. (A) Distribution of serum galectin-3 (B) Comparison of LVG geometry by galectin-3 quartiles.
