# Supplementary material for: Galectin-3 as a prognostic biomarker in haemodialysis patients with preserved or mildly reduced ejection fraction
Source: Clin Kidney J. 2025 Oct 7;18(10):sfaf306. doi: 10.1093/ckj/sfaf306 (PMC12556406; doi:10.1093/ckj/sfaf306)
Supplement: sfaf306_Supplemental_Files [file sfaf306_supplemental_files.zip › Supplemental Table 1 R1.docx]

**Supplemental Table 1. Cause of death and corresponding galectin-3 levels (ng/mL)**

| **Cause of death** | **N** | **Median galectin-3** | **IQR** |
| --- | --- | --- | --- |
| **CV deaths** | 30 |  |  |
| MI/SCD | 10 | 49.7 | 38.4-63.2 |
| HF-related | 10 | 47.1 | 34.3-53.5 |
| Cerebrovascular accident-related | 10 | 37.3 | 26.5-39.4 |
| **Non-CV deaths** | 54 |  |  |
| Infection | 38 | 38.0 | 24.3-46.3 |
| Malignancy | 8 | 42.8 | 22.3-68.3 |
| Others | 8 | 43.4 | 30.9-63.5 |
